# The GUSBP11/miR-29c-3p axis facilitates gastric cancer malignancy through ceRNA mechanism

**DOI:** 10.1186/s41065-026-00719-1

**Published:** 2026-08-12

**Authors:** Yahan Zhao, Chan Lv, Jincai Chen, Xiaohui Wang, Riwei Wang

**Affiliations:** 1 Department of Gastroenterology, The First Affiliated Hospital of Xi’an Jiaotong University, Xi’an, 710061 China; 2 Department of Gastroenterology, Shuguang Hospital Affiliated to Shanghai University of Traditional Chinese Medicine, Shanghai, 201203 China; 3 Department of Gastrointestinal Surgery, Affiliated Hospital of Jiangsu University, Zhenjiang, 212001 China; 4 Department of Hernia and Abdominal Wall Surgery, Ganzhou Hospital-Nanfang Hospital, Southern Medical University (Ganzhou People’s Hospital), Ganzhou, 341000 China; 5 Three Branches of General Surgery, Jiujiang City Key Laboratory of Cell Therapy, Jiujiang NO.1 People’s Hospital, No. 48, Talingnan Road, Jiujiang, 332000 China

**Keywords:** Gastric cancer, GUSBP11, miR-29c-3p, ceRNA, EMT

## Abstract

**Background:**

Long non-coding RNAs (lncRNAs) function as pivotal regulators in gastric cancer (GC) pathogenesis via competitive endogenous RNA (ceRNA) networks. However, the precise role of GUSBP11 in GC progression remains inadequately characterized.

**Aim:**

This investigation aimed to delineate the biological function of GUSBP11 in GC and to ascertain whether it regulates malignant phenotypes via miR-29c-3p.

**Methods:**

GUSBP11, miR-29c-3p, and THBS2 expression levels were quantified in tissues and cells using RT-qPCR. Prognostic value of GUSBP11 in GC was assessed via Kaplan-Meier curve and Cox analysis. In vitro, AGS and HGC-27 cell lines were employed for loss- and gain-function assays. Cellular viability was measured via CCK-8, while migration and invasion capacities were examined using Transwell chambers. Epithelial-mesenchymal transition (EMT) markers were detected by RT-qPCR. Mechanistic interplay was validated through dual-luciferase reporter assays and miRNA inhibitor rescue experiments.

**Results:**

GUSBP11 exhibited upregulation in GC tissues and cell lines, correlating significantly with lower 5-year overall survival. Functional assays demonstrated that GUSBP11 overexpression enhanced viability, migration, and invasion, whereas its knockdown elicited the opposite phenotype. Mechanistically, GUSBP11 operated as a ceRNA by sponging miR-29c-3p, thereby relieving suppression of downstream effector THBS2. Co-transfection with a miR-29c-3p inhibitor effectively reversed the tumor-suppressive consequences of GUSBP11 silencing, restoring THBS2 expression and EMT programming.

**Conclusion:**

The GUSBP11/miR-29c-3p axis exerts a pro-oncogenic influence on GC progression. This regulation offer fresh insights into GC pathophysiology and identifies prospective biomarkers for therapeutic intervention.

**Supplementary Information:**

The online version contains supplementary material available at https://doi.org/10.1186/s41065-026-00719-1.

## Introduction

Gastric cancer (GC) constitutes one of the most prevalent gastrointestinal malignancies globally, with its incidence and mortality rates persistently occupying prominent positions among all neoplasms [1, 2]. Despite notable advances in surgical, adjuvant chemoradiotherapy, and immune-targeted interventions over recent decades, the overall prognosis for individuals afflicted with GC remains far from satisfactory [3, 4]. This therapeutic impasse is predominantly attributable to the insidious onset of the disease, suboptimal rates of early detection, pronounced invasive and metastatic propensities, and elevated recurrence frequency [5, 6]. Recent studies have further elucidated the multifaceted molecular mechanisms underlying of GC, revealing that dysregulated epigenetic modifications such as promoter hypomethylation-mediated oncogene activation [7], aberrant cell death modalities encompassing apoptosis, ferroptosis, and necroptosis that modulate therapeutic resistance and metastatic dissemination [8–10]. Furthermore, remodeling of the immune microenvironment via CD8 T cell-mediated antitumor immunity and cGAS-STING signaling [11, 12], and metabolic reprogramming involving lipid homeostasis disruption [10] collectively contribute to GC progression and heterogeneity. Consequently, deciphering the molecular mechanisms governing GC onset and metastatic progression, alongside the identification of novel diagnostic biomarkers and exploitable therapeutic targets, holds profound clinical significance.

Long non-coding RNAs (lncRNAs), have now been substantiated to participate extensively in diverse aspects of gene regulation, encompassing epigenetic modification, transcriptional regulation, and post-transcriptional processing [13–15]. Particularly noteworthy is the competing endogenous RNA (ceRNA) hypothesis, which proffers a novel mechanistic vista for lncRNA functionality [16]. According to this framework, lncRNAs may bind to miRNAs and alleviate downstream target mRNAs, thereby constituting an intricate lncRNA-miRNA-mRNA regulatory network [17]. In recent years, an expanding network of ceRNA has been systematically identified in GC [18, 19]. For instance, PVT1 enhances proliferation, invasion, and cell cycle progression in GC by binding to miR-16 and thereby relieving its inhibition of CCND1 [20]. MIR181A2HG modulates miR-5680 via a ceRNA axis, leading to elevated VCAN, which subsequently facilitates lymphatic metastasis in GC [21]. These findings open up new avenues for personalized targeted therapy for GC.

Recent investigations have revealed that GUSBP11 exhibits notably overexpression across a spectrum of human malignancies and exerts pro-tumorigenic functions via ceRNA networks. For example, in nasopharyngeal carcinoma, GUSBP11 sponges miR-1226-3p to elevate TM9SF4 abundance, thereby accelerating proliferation and metastasis [22]. Nevertheless, the biological effects and potential mechanisms of GUSBP11 in GC remain entirely uncharted, warranting systematic research. Concurrently, a mounting body of evidence attests that miR-29c-3p is appreciably downregulated of diverse solid tumors, functioning as a tumor suppressor. Within the GC context, miR-29c-3p levels are markedly diminished [23]. Functional studies further corroborate that inhibited miR-29c-3p expression substantially enforces GC cell proliferation and metastasis [24, 25]. However, whether miR-29c-3p is regulated by GUSBP11 remains to be elucidated. THBS2, a stromal-derived glycoprotein, participates in various physiological processes encompassing cell-matrix interactions, angiogenesis, and tissue remodeling [26–28]. Multiple lines have confirmed that THBS2 is conspicuously overexpressed in GC tissues and may play an oncogenic role [29, 30]. Nonetheless, the regulatory mechanisms of THBS2 expression in GC, particularly whether it is governed by ceRNA networks, remain unclear.

The present investigation was to systematically delineate expression profile and clinical significance of GUSBP11 in GC, and to ascertain its regulatory influence upon GC cell behavior. Furthermore, we aimed to verify whether GUSBP11 functions as a molecular sponge for miR-29c-3p, thereby alleviating THBS2 inhibition and culminating in a GUSBP11/miR-29c-3p/THBS2 ceRNA axis. Collectively, this study is expected to provide novel mechanistic insights into GC and identify potential therapeutic targets.

## Materials and methods

### Clinical specimen

GC tissues (Tumor) and corresponding adjacent non-neoplastic mucosae (Normal) were procured as paired specimens from 133 patients undergoing curative resection at Ganzhou Hospital-Nanfang Hospital, Southern Medical University between 2018 and 2020. Patients with prior chemotherapy, radiotherapy, targeted therapy, history of other malignancies, incomplete clinicopathological data, or stage IV disease were excluded. Histopathological diagnosis was independently verified by two board-certified pathologists according to the WHO classification. All specimens were snap-frozen in liquid nitrogen and archived at − 80 °C until subsequent analyses. Clinicopathological characteristics encompass age, gender, tumor size, histological grade, TNM stage, and nodal status were collated from electronic medical records. Postoperative follow-up data were acquired through outpatient revisits and telephone interviews, with a follow-up period of 60 months. The study protocol received endorsement from the Institutional Ethics Committee of Ganzhou Hospital-Nanfang Hospital, Southern Medical University, and written informed consent was secured from every participant prior to tissue acquisition.

### Cell culture

Human GC cell lines (AGS, SNU-1, MKN-45, HGC-27) and the immortalized gastric epithelial cell line GES-1 were sourced from ATCC (Manassas, VA, USA). AGS and SNU-1 were propagated in RPMI-1640 medium (Gibco, Grand Island, NY, USA), whereas HGC-27, MKN45, and GES-1 were cultivated in DMEM (Gibco). All media were augmented with 10% FBS (HyClone, Logan, UT, USA) and 1% penicillin-streptomycin solution. Cells were housed in an incubator at 37 °C under 5% CO₂. Upon attaining approximately 80% confluence, cultures were dissociated using 0.25% trypsin-EDTA (Gibco) and sub-cultured at a ratio of 1:3 to 1:5. Logarithmic-phase cells were employed for all subsequent experiments.

### Oligonucleotide transfection

Small interfering RNA specifically targeting GUSBP11 (si-GUSBP11), negative control (si-NC), GUSBP11 overexpression plasmid (oe-GUSBP11), empty vector control (oe-NC), miR-29c-3p inhibitor, and corresponding inhibitor NC were procured from GenePharma (Shanghai, China). Transient transfection was executed using Lipofectamine 3000 reagent (Invitrogen, Carlsbad, CA, USA) in strict accordance with manufacturer’s protocol. Cells were harvested 48 h post-transfection for subsequent analyses, and knockdown or overexpression efficacy was verified via RT-qPCR prior to functional assessments.

### RNA extraction and RT-qPCR

Total RNA was isolated from tissue specimens and cells utilizing TRIzol reagent (Invitrogen, Carlsbad, CA, USA). For mRNA and lncRNA detection, cDNA was synthesized employing the PrimeScript RT Master Mix (Takara, Kusatsu, Japan). For miRNA quantification, cDNA was generated using the Mir-X miRNA First-Strand Synthesis Kit (Takara). qPCR was conducted on an ABI 7500 Fast System (Applied Biosystems, Foster City, CA, USA) using SYBR Premix Ex Taq II (Takara). GAPDH served as endogenous reference for GUSBP11, THBS2, E-cadherin, N-cadherin, and vimentin, whereas U6 was utilized for miR-29c-3p normalization. Relative expression levels were computed via the 2^−ΔΔCt^ method.

### Cell viability assays

Transfected AGS and HGC-27 cells were seeded into 96-well plates at a density of 2 × 10³ cells per well. Following adherence overnight, cellular viability was monitored at predetermined intervals (0, 24, 48, 72 h). At each time point, 10 µL of CCK-8 reagent (Dojindo, Kumamoto, Japan) was dispensed into each well, and plates were incubated for an additional 2 h. Optical density was quantified at 450 nm employing a microplate spectrophotometer.

### Migration and invasion evaluation

For invasion assays, the upper chamber membranes were pre-coated with Matrigel matrix (BD, San Jose, CA, USA). Transfected AGS and HGC‑27 cells were harvested, resuspended in 200 µL of serum-free medium, and seeded into the upper chamber (Corning, NY, USA) at a density of 5 × 10⁴ cells per well. The lower chamber (Corning) was replenished with 600 µL medium containing FBS. Cells that had traversed the membrane were immobilized with 4% paraformaldehyde, stained with 0.1% crystal violet solution, and visualized under an inverted light microscope.

### Subcellular fractionation and localization analysis

To ascertain the subcellular distribution of GUSBP11, nuclear and cytoplasmic RNAs were isolated from AGS and HGC-27 cells using the PARIS Kit (Thermo Fisher, Waltham, MA, USA). RT-qPCR was subsequently performed to quantify GUSBP11 abundance in each fraction. GAPDH served as the cytoplasmic marker, whereas U6 was utilized as the nuclear marker.

### Candidate miRNA screening and selection

Candidate miRNAs potentially interacting with GUSBP11 were identified from the starBase/ENCORI database using the miRNA-lncRNA interaction module. miRNAs supported by Ago-CLIP evidence and multiple binding sites were initially considered. These candidates were subsequently screened through a stepwise selection strategy based on the following criteria: (i) reported tumor-suppressive roles in GC or other malignancies; (ii) inverse expression profile, meaning downregulation in GC tissues while GUSBP11 upregulation; (iii) functional involvement in EMT, migration, and invasion pathways; and (iv) confirmed association with unfavorable prognosis in GC. Among the eligible candidates, miR-29c-3p met all criteria and was therefore selected for further experimental verification.

### Dual-luciferase reporter assay

Wild-type (wt) and mutant (mut) fragments encompassing predicted miR-29c-3p recognition sequences were synthesized and cloned into the psiCHECK-2 vector (Promega, Madison, WI, USA). For luciferase activity quantification, cells were seeded into 24-well plates and co-transfected with the respective reporter plasmid and miR-29c-3p mimic or mimic NC utilizing Lipofectamine 3000 reagent. At 48 h post-transfection, cells were harvested and luciferase activities were measured employing the Dual-Luciferase Reporter Assay System (Promega).

### Western blot analysis

Total protein was extracted from cells employing RIPA lysis buffer (Beyotime, Shanghai, China) augmented with protease and phosphatase inhibitor (Roche, Basel, Switzerland). Protein concentration was quantified via the BCA assay kit (Beyotime). Protein was fractionated on 8% SDS-PAGE gels and subsequently transferred to PVDF membranes (Millipore, Billerica, MA, USA). Membranes were blocked with 5% non-fat milk in TBST for 1 h at room temperature and then incubated overnight at 4 °C with primary antibodies against THBS2 (Abcam, Cambridge, UK) and GAPDH (Abcam). Following three washes with TBST, membranes were incubated with HRP-conjugated secondary antibodies (Abcam) for 1 h. Protein bands were visualized using an enhanced chemiluminescence (ECL) substrate and imaged with a ChemiDoc XRS+ system. Band intensities were quantified using ImageJ software, and relative protein expression was normalized to GAPDH.

### Statistical analysis

All statistical evaluations were employed via GraphPad Prism 9.0 and SPSS 27.0. Data are presented as mean ± SD from a minimum of three independent experiments. For comparisons of two discrete groups, Student’s t-test was applied; for multiple group contrasts, one-way ANOVA was employed. Associations between GUSBP11 and clinicopathological features were scrutinized via chi-square test. Survival curves were constructed utilizing the Kaplan-Meier methodology. Prognostic determinants were identified through multivariate Cox proportional hazards regression models simultaneously incorporating GUSBP11 expression alongside age, gender, tumor size, differentiation, TNM stage, and lymph node metastasis. *P* value inferior to 0.05 was regarded as statistically significant.

## Results

### GUSBP11 exhibits marked overexpression in GC and correlates with adverse clinicopathological features

**Fig. 1:**
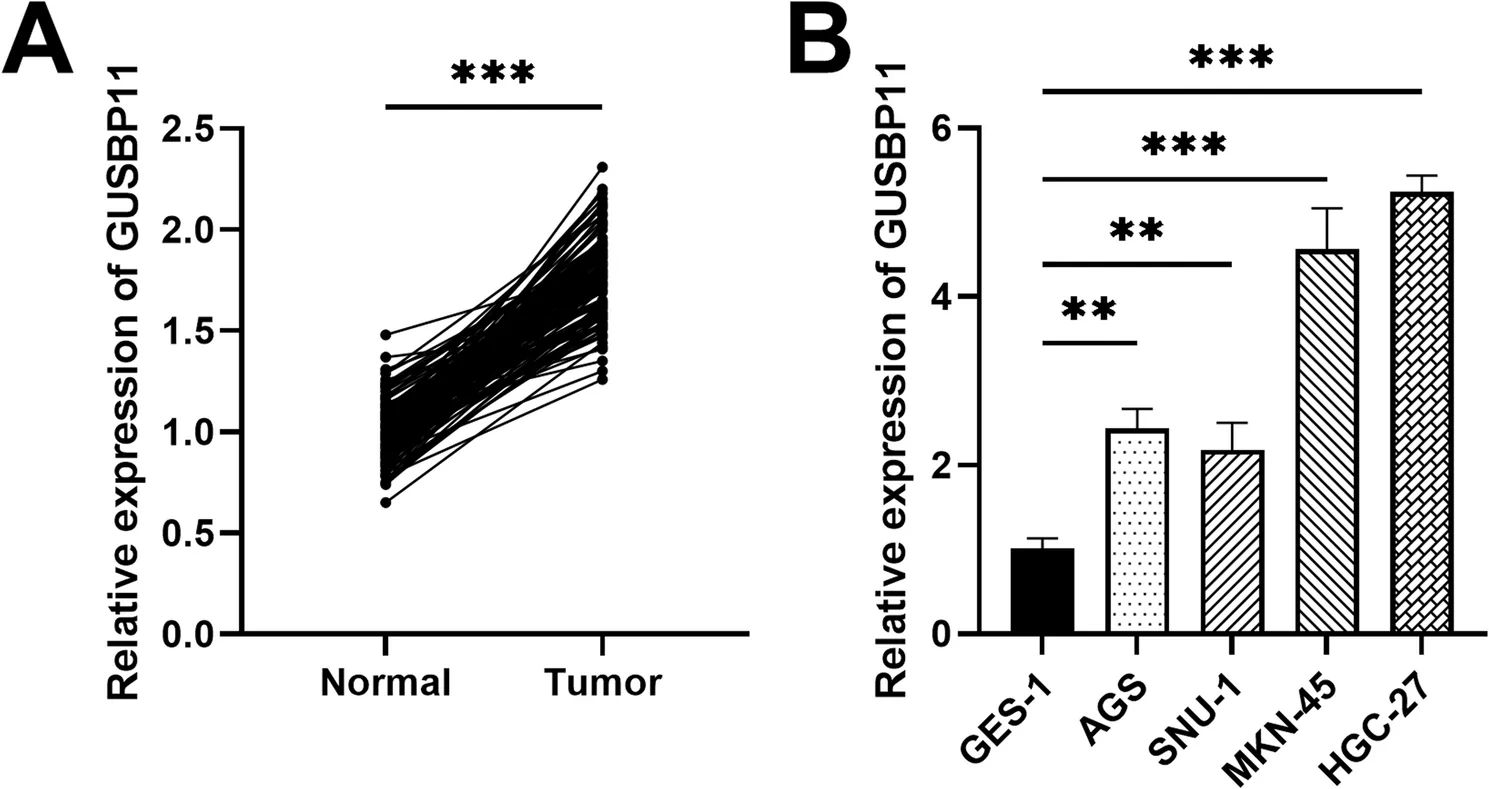
GUSBP11 exhibits marked overexpression in GC. **A** Relative GUSBP11 abundance in 133 paired GC tissues (Tumor) and adjacent non-neoplastic mucosae (Normal). **B** GUSBP11 expression across GC cell lines (AGS, SNU-1, MKN-45, HGC-27) relative to the immortalized gastric epithelial cell line GES-1. **P < 0.01, ***P < 0.001

To ascertain GUSBP11 expression in GC, RT-qPCR was initially deployed across 133 paired tumor and normal tissue specimens. A conspicuous elevation of GUSBP11 was observed in tumor tissues (Fig. 1A). Concordantly, GC cell lines (AGS, SNU-1, MKN-45, HGC-27) revealed substantially elevated GUSBP11 abundance compared with the immortalized gastric epithelial cell line GES-1, with metastatic lines (MKN-45, HGC-27) exhibiting the most pronounced upregulation (Fig. 1B). To further explore the clinical implications of GUSBP11 dysregulation, the patient cohort was stratified into high (*n* = 72) and low (*n* = 61) expression groups based on average tumor GUSBP11 value. As delineated in Table 1, heightened GUSBP11 expression displayed a significant association with poor differentiation (*P* = 0.022), advanced TNM stage (*P* = 0.004), and positive lymph node metastasis (*P* = 0.018). In contrast, no substantive correlations were observed with age, gender, or tumor size (*P* > 0.05).

**Table 1:** Correlation between GUSBP11 expression and clinicopathological features of GC patients

		Total cases (*n* = 133)	GUSBP11 expression		*P* value
			High (*n* = 72)	Low (*n* = 61)	
Age	≤ 60	64	36	28	0.637
	> 60	69	36	33	
Gender	Male	89	49	40	0.762
	Female	44	23	21	
Tumor size (cm)	≤ 5	77	38	39	0.194
	> 5	56	34	22	
Differentiation	Well/Moderate	62	27	35	0.022
	Poor	71	45	26	
TNM stage	Ⅰ-Ⅱ	90	41	49	0.004
	Ⅲ	43	31	12	
Lymph node metastasis	Negative	68	30	38	0.018
	Positive	55	42	23	

*TNM* tumor-node-metastasis

### Elevated GUSBP11 portends unfavorable prognosis in GC

**Fig. 2:**
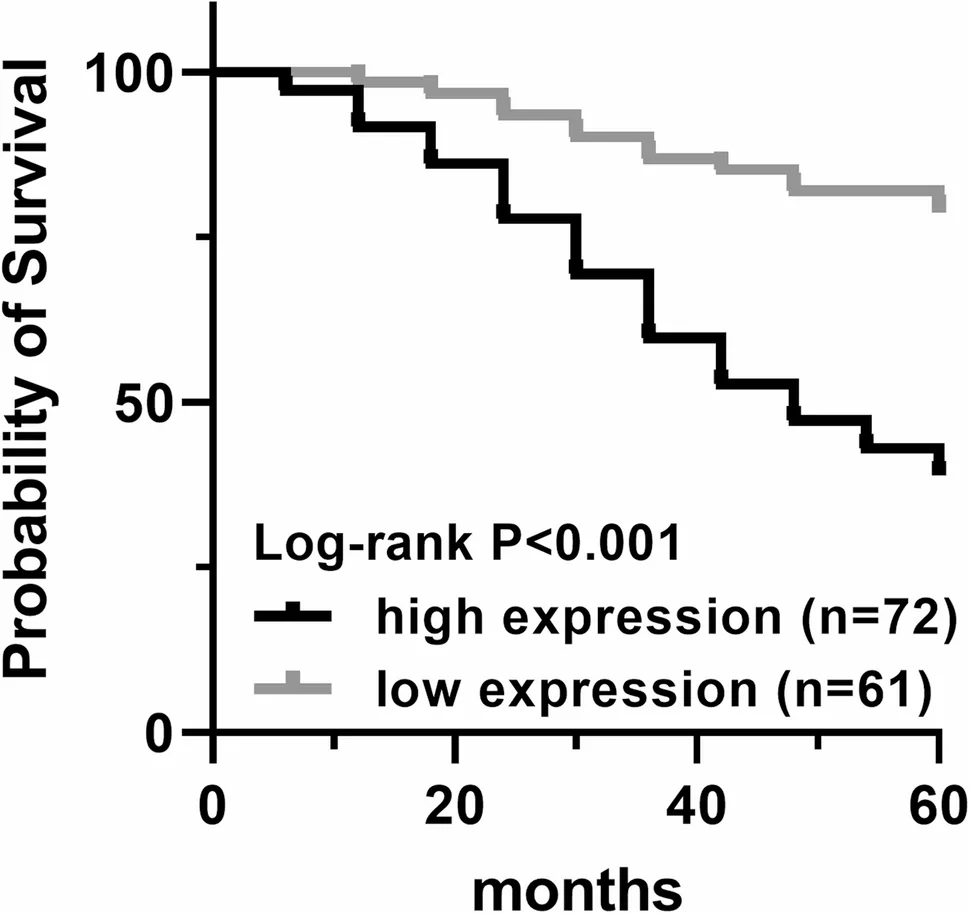
Elevated GUSBP11 portends unfavorable prognosis in GC. Kaplan-Meier overall survival curves for GC patients

To appraise prognostic implications of GUSBP11 dysregulation, Kaplan-Meier survival curves were constructed for the 133-patient cohort stratified by the average tumor GUSBP11 expression. As illustrated in Fig. 2, individuals harboring high GUSBP11 levels endured a conspicuously abbreviated 5-year overall survival (log‑rank *P* < 0.001). To ascertain whether GUSBP11 constitutes an independent prognostic factor, multivariate Cox analyses were undertaken with simultaneous adjustment for age, gender, tumor size, differentiation, TNM stage, and lymph node metastasis. Multivariate results summarized in Table 2, after adjustment for these covariates, GUSBP11 overexpression retained independent prognostic significance (HR = 2.588, 95% CI:1.347–4.975, *P* = 0.004), alongside differentiation (HR = 1.881, 95% CI:1.072–3.302, *P* = 0.028), TNM stage (HR = 2.006, 95% CI:1.158–3.474, *P* = 0.013), and lymph node metastasis (HR = 1.872, 95% CI:1.079–3.247, *P* = 0.026).

**Table 2:** Cox proportional hazards regression analysis of overall survival of GC patients

	HR	95% CI		*P* value
		Lower	Upper	
GUSBP11	2.588	1.347	4.975	0.004
Age	1.044	0.608	1.793	0.877
Gender	1.254	0.697	2.253	0.450
Tumor size	1.344	0.774	2.335	0.293
Differentiation	1.881	1.072	3.302	0.028
TNM stage	2.006	1.158	3.474	0.013
Lymph node metastasis	1.872	1.079	3.247	0.026

*TNM* tumor-node-metastasis

### GUSBP11 potentiates GC cell viability, migration, invasion, and EMT

**Fig. 3:**
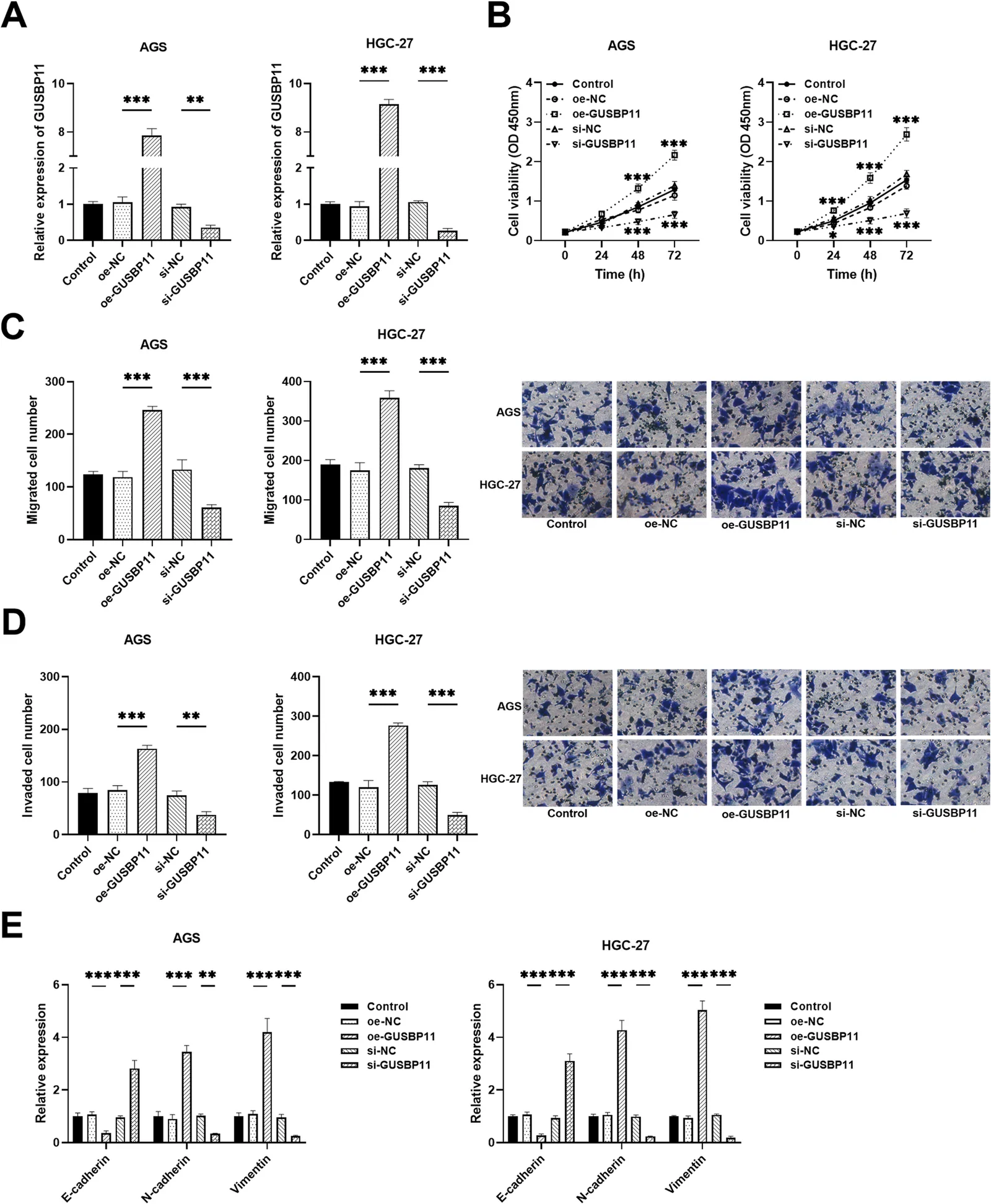
GUSBP11 potentiates GC cell viability, migration, invasion, and EMT. **A** RT-qPCR corroboration of oe-GUSBP11 and si-GUSBP11 transfection efficiency in AGS and HGC-27 cells. **B** Cell viability assays following GUSBP11 overexpression or silencing. **C**, **D** Representative Transwell migration and invasion images and quantification following GUSBP11 overexpression or silencing. **E** Relative mRNA levels of EMT markers (E-cadherin, N-cadherin, Vimentin) following GUSBP11 overexpression or silencing. **P < 0.01, ***P < 0.001

To elucidate functional contributions of GUSBP11 to GC progression, gain- and loss-function assays were conducted in AGS and HGC-27 cells. Transfection efficacy was corroborated via RT-qPCR, which verified pronounced GUSBP11 upregulation in oe-GUSBP11 groups and downregulation in si-GUSBP11 groups relative to their respective controls (Fig. 3A). CCK-8 assays revealed that enforced GUSBP11 expression markedly augmented cell viability, whereas GUSBP11 silencing attenuated cell viability in both cell lines (Fig. 3B). Transwell assessments further disclosed that GUSBP11 overexpression propelled migratory and invasive propensities, while GUSBP11 silencing exerted pronounced inhibitory effects (Fig. 3C, D). In concordance with these phenotypic alterations, GUSBP11 upregulation diminished E-cadherin abundance while elevating N-cadherin and Vimentin levels. Reciprocally, GUSBP11 knockdown enhanced E-cadherin expression and suppressed N-cadherin and Vimentin levels (Fig. 3E).

### GUSBP11 as a molecular sponge for miR-29c-3p

**Fig. 4:**
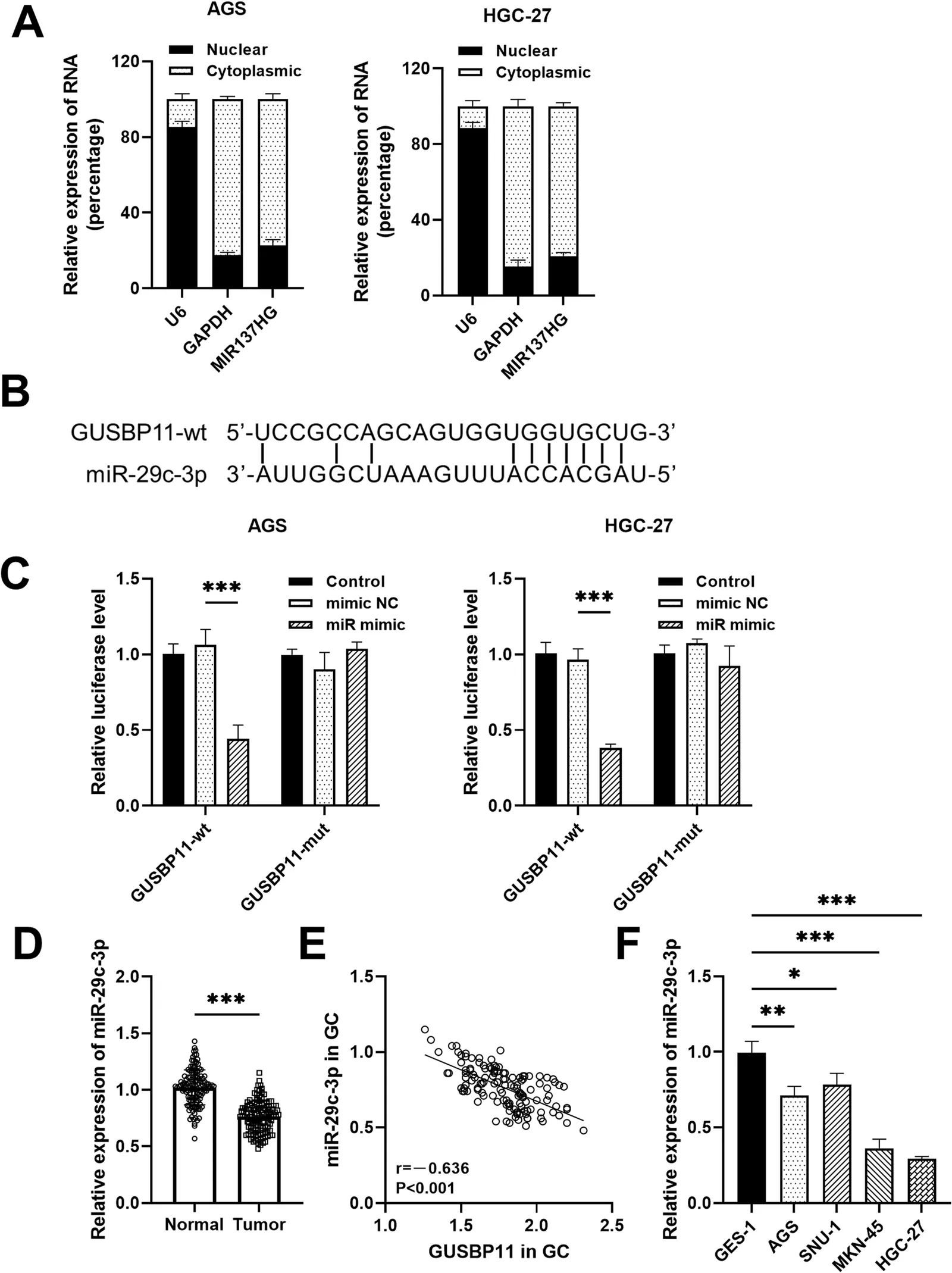
GUSBP11 as a molecular sponge for miR-29c-3p. **A** Subcellular localization of GUSBP11. **B** Schematic depiction of the predicted complementary binding interface between GUSBP11 and miR-29c-3p. **C** Dual-luciferase reporter assay co-transfected with miR-29c-3p mimic and GUSBP11-wt or GUSBP11-mut reporter plasmids. **D** Relative miR-29c-3p expression in 133 paired GC tissues (Tumor) and adjacent non-neoplastic mucosae (Normal). **E** Pearson correlation analysis between GUSBP11 and miR-29c-3p in GC specimens. **F** miR-29c-3p abundance across GC cell lines relative to GES-1. *P < 0.05, **P < 0.01, ***P < 0.001

Subcellular fractionation coupled with RT-qPCR revealed that GUSBP11 was predominantly enriched in the cytoplasmic compartment of both AGS and HGC-27 cells (Fig. 4A). Bioinformatic analysis forecasted a putative complementary binding interface between GUSBP11 and the seed region of miR-29c-3p (Fig. 4B). Co-transfection of miR-29c-3p mimic with a GUSBP11-wt reporter construct elicited a pronounced reduction in luciferase activity, whereas co-transfection with GUSBP11-mut eliminated this suppressive effect (Fig. 4C). RT-qPCR assessment divulged that miR-29c-3p levels were substantially diminished in GC tissues (Fig. 4D). Furthermore, Pearson correlation analysis revealed an inverse association between GUSBP11 and miR-29c-3p in GC specimens (*r* = − 0.636, *P* < 0.001, Fig. 4E). This provides clinical support for the proposed ceRNA interaction at the GUSBP11/miR-29c-3p axis. Congruently, a similar expression gradient was observed across GC cell lines, wherein metastatic lines (MKN-45, HGC-27) exhibited the most pronounced miR-29c-3p reduction relative to GES-1 (Fig. 4F).

### miR-29c-3p inhibition reverses GUSBP11 silencing-mediated tumor suppression

**Fig. 5:**
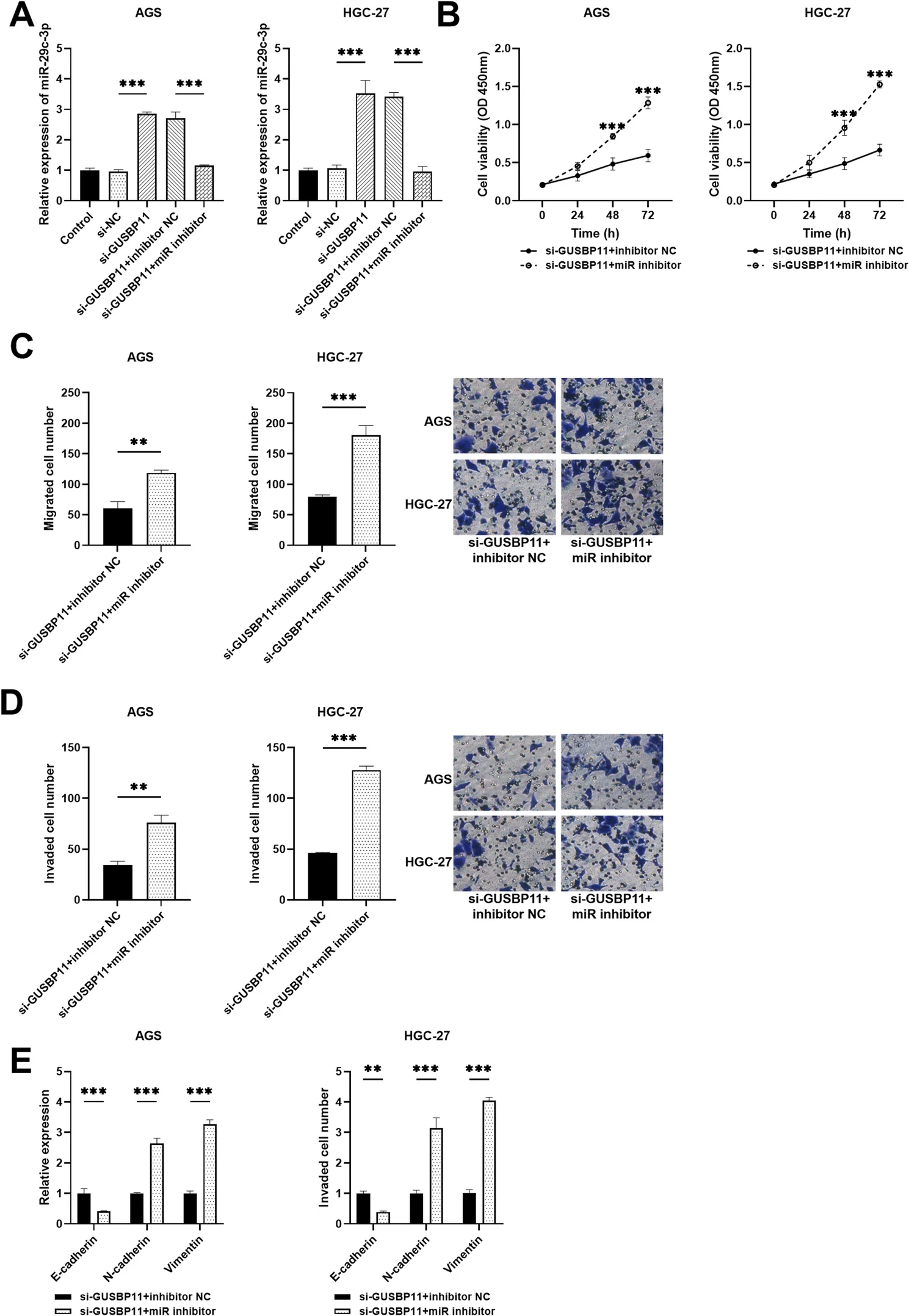
miR-29c-3p inhibition reverses GUSBP11 silencing-mediated tumor suppression. **A** RT-qPCR validation of miR-29c-3p levels in AGS and HGC-27 cells co-transfected with si-GUSBP11 and miR-29c-3p inhibitor. **B** Cell viability assays following co-transfected with si-GUSBP11 and miR-29c-3p inhibitor. **C**, **D** Representative Transwell migration and invasion images and quantification following co-transfected with si-GUSBP11 and miR-29c-3p inhibitor. **E** mRNA expression of EMT markers following co-transfected with si-GUSBP11 and miR-29c-3p inhibitor. **P < 0.01, ***P < 0.001

To ascertain whether the tumor-suppressive effects of GUSBP11 knockdown are dependent on miR-29c-3p, rescue experiments were designed via co-transfection of si-GUSBP11 with either a miR-29c-3p inhibitor or inhibitor NC. RT-qPCR verification corroborated that miR-29c-3p abundance was elevated in the si-GUSBP11 cohort, whereas markedly diminished in the si-GUSBP11 + miR-29c-3p inhibitor cohort (Fig. 5A). CCK-8 assays divulged that the attenuated proliferative capacity elicited by GUSBP11 knockdown was restored upon miR-29c-3p inhibition (Fig. 5B). Additionally, Transwell evaluations demonstrated that co-transfection with miR-29c-3p inhibitor reversed the suppression of migratory and invasive propensities caused by GUSBP11 silencing (Fig. 5C, D). Furthermore, RT-qPCR analyses of EMT markers revealed that miR-29c-3p inhibition reinstated the mesenchymal phenotype, as evidenced by diminished E-cadherin levels concurrent with elevated N-cadherin and Vimentin expression (Fig. 5E).

### THBS2 is a direct downstream effector of the GUSBP11/miR-29c-3p axis

**Fig. 6:**
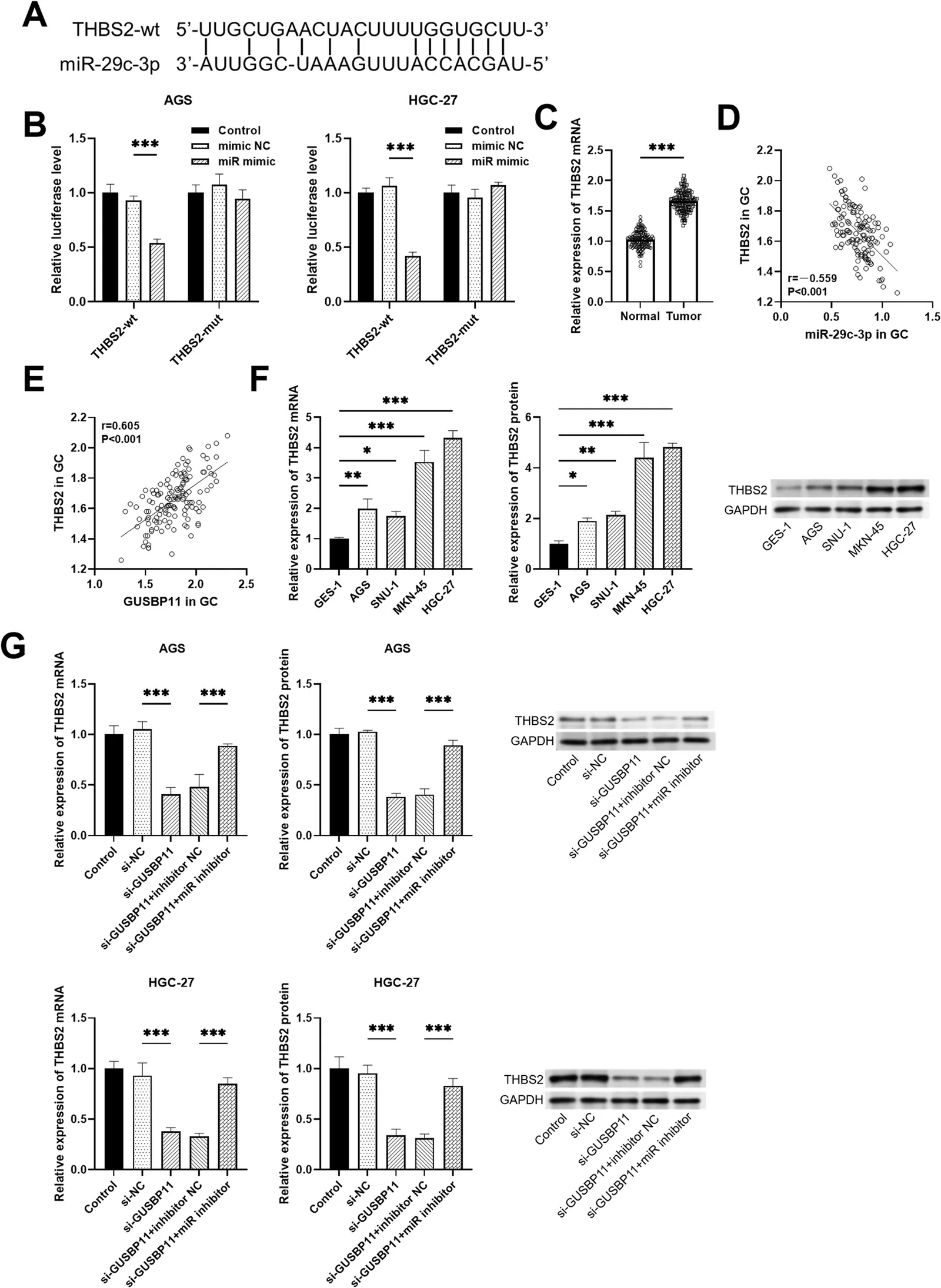
THBS2 is a direct downstream effector of the GUSBP11/miR-29c-3p axis. **A** Schematic depiction of the predicted complementary binding interface between miR-29c-3p and THBS2. **B** Dual-luciferase reporter assay co-transfected with miR-29c-3p mimic and THBS2-wt or THBS2-mut reporter plasmids. **C** Relative THBS2 expression in 133 paired GC tissues (Tumor) and adjacent non-neoplastic mucosae (Normal). **D**, **E** Pearson correlation analysis between THBS2 and miR-29c-3p or GUSBP11 in GC specimens. **F** THBS2 mRNA and protein levels across GC cell lines relative to GES-1. **G** THBS2 mRNA and protein levels following transfected with si-GUSBP11 or co-transfected with miR-29c-3p inhibitor. *P < 0.05, **P < 0.01, ***P < 0.001

Bioinformatic analysis forecasted a conserved complementary binding interface of miR-29c-3p and the 3’-UTR of THBS2 mRNA (Fig. 6A). Co-transfection of miR-29c-3p mimic with THBS2-wt elicited a pronounced decrease in luciferase activity, whereas co-transfection with THBS2-mut abrogated this repressive effect (Fig. 6B). Moreover, RT-qPCR disclosed that THBS2 mRNA abundance were conspicuously elevated in GC tissues (Fig. 6C). Pearson correlation analysis revealed a negative association between THBS2 and miR-29c-3p (*r* = − 0.559, *P* < 0.001, Fig. 6D), alongside a positive correlation between THBS2 and GUSBP11 (*r* = 0.605, *P* < 0.001, Fig. 6E) in GC specimens. These clinical associations, combined with the negative correlation between GUSBP11 and miR-29c-3p, suggest that these three molecules are closely interconnected in GC tissues through a ceRNA regulatory network. Concordant with this observation, GC cell lines exhibited markedly increased THBS2 mRNA and protein levels (Fig. 6F). Furthermore, in AGS and HGC-27 cells, silencing GUSBP11 notably reduced THBS2 mRNA and protein levels, whereas co-transfection with a miR-29c-3p inhibitor restored these levels (Fig. 6G).

## Discussion

GC stands as one of the most prevalent malignancies afflicting digestive tract, with its pronounced invasiveness and persistently dismal clinical prognosis posing serious challenges to contemporary oncological management [31]. Consequently, elucidation of the molecular mechanisms propelling GC progression, together with the identification of novel targets harboring both diagnostic utility and therapeutic purposes, carries profound translational import. The present investigation is the first systematic delineation of GUSBP11 expression characteristics, clinicopathological associations, and biological functions in GC. Moreover, it unveiled a mechanism that GUSBP11 operates as a ceRNA by binding to miR-29c-3p, thereby derepressing THBS2 expression. These findings proffer fresh experimental substantiation for the molecular pathology of gastric cancer and may ultimately inform the development of incipient diagnostic and therapeutic modalities. This provides new experimental evidence for molecular pathological research on GC.

The principal revelation of this investigation was that GUSBP11 upregulation across GC tissues and cell lines, with its augmented abundance exhibiting robust correlations with adverse clinicopathological indices. Furthermore, statistical analysis corroborated that patients harboring elevated GUSBP11 have markedly shorter five-year overall survival, and multivariate analysis substantiated GUSBP11 as an independent prognostic factor for GC. These observations are consistent with the documented oncogenic attributes of GUSBP11 in disparate malignancies, including nasopharyngeal carcinoma and lung adenocarcinoma [22, 32], suggesting that this lncRNA may serve a conserved pro-tumorigenic function across a pan-cancer spectrum. It is worth emphasis, however, that antecedent investigations have largely confined to descriptive expression profiling within single cancer types, seldom addressing into the downstream regulatory by GUSBP11. Therefore, the present work partially redresses this shortcoming by providing a more detailed mechanistic delineation.

At the functional level, gain- and loss-function experiments demonstrated that GUSBP11 substantially enhanced GC cell viability, migration and invasion capacity, concomitant with induction of EMT, as evinced by diminished E-cadherin abundance alongside elevated N-cadherin and Vimentin levels. EMT is a critical step in which epithelial tumor cells acquire a mesenchymal phenotype and initiate local invasion and distant metastasis [33, 34]. The present findings intimate that GUSBP11 may promote the malignant progression of GC by driving EMT. Of particular note, HGC-27 cells derived from a lymph node metastatic deposit exhibited higher GUSBP11 expression and greater responsiveness to silencing intervention relative to AGS cells from primary tumor. This difference aligns with the gradation of malignancy between the two cell lines and is consistent with previous studies on other lncRNAs in GC [35, 36]. Such concordance further reinforces the positive correlation between GUSBP11 abundance and the invasive potential of GC cells.

The ceRNA hypothesis constitutes a key theoretical framework for elucidating the mechanism of lncRNA in recent years [37, 38]. In this work, predictions combined with dual-luciferase reporter assays confirmed a direct binding interface of GUSBP11 and miR-29c-3p. miR-29c-3p has been substantiated to exert tumor-suppressive functions across various malignancies, including lung cancer, omental cancer, and colorectal cancer [39–41]. Our data showed that miR-29c-3p abundance is markedly reduced in GC tissues and exhibits an inverse correlation with GUSBP11, suggesting that GUSBP11 may attenuate miR-29c-3p via a molecular sponge. Rescue experiments further unveiled that targeted inhibition of miR-29c-3p substantially reversed the proliferative inhibition, migratory and invasive attenuation, and EMT reversal elicited by GUSBP11 knockdown. This functionally confirms that the oncogenic effect of GUSBP11 depends, at minimum partially, on its downregulation of miR-29c-3p.

THBS2 emerged as a downstream effector of miR-29c-3p in this study. While prior investigations have largely centered upon its participation in extracellular matrix remodeling and angiogenesis regulation [42, 43]. Recent reports document THBS2 overexpression in colorectal and non-small cell lung cancer, where it portends unfavorable prognosis [44, 45]. Given that THBS2 is a secreted matricellular protein abundantly deposited in the extracellular matrix, it is plausible that elevated THBS2 facilitates GC invasion through MMP2/MMP1-mediated matrix degradation and invadopodia stabilization [46–48]. Moreover, THBS2 may reinforce EMT programming via interaction with TGF-β signaling, thereby maintaining the invasive phenotype [49]. Secreted THBS2 may also contribute to pre-metastatic niche formation at distant sites [50]. Although these putative mechanisms require further validation, they link the GUSBP11/miR-29c-3p/THBS2 axis to established metastasis pathways. The present work not only corroborated elevated THBS2 abundance in GC tissues and cell lines, but further revealed an inverse correlation between THBS2 and miR-29c-3p, alongside a positive association with GUSBP11. These three genes form a complete ceRNA regulatory axis. This finding provides a mechanism for THBS2 dysregulation in GC and broadens the profile of downstream effector molecules in the GUSBP11/miR-29c-3p signaling pathway.

Of course, this study also has several limitations. First, due to limitations in clinical sample size and the single-center origin, the prognostic value of GUSBP11 still needs to be validated in larger and multicenter cohorts. Second, due to limitations in experimental conditions, this study was unable to conduct in vivo experiments, and the actual regulatory efficacy of the GUSBP11/miR-29c-3p/THBS2 axis in the in vivo microenvironment remains to be further investigated. In addition, the specific signaling pathways activated by the downstream of THBS2 have not been deeply explored, and subsequent studies will be expanded around the above issues.

Overall, this study provides the first evidence that GUSBP11 is highly expressed in GC and associated with unfavorable prognosis. GUSBP11 upregulates THBS2 expression by binding to miR-29c-3p, thereby facilitating GC cell viability, invasion, and EMT. The discovery of GUSBP11/miR-29c-3p/THBS2 regulatory axis provides a new perspective on understanding molecular mechanisms of GC and lays a preliminary theoretical foundation for the development of future targeted therapeutic strategies.

## Supplementary Information

Supplementary Material 1.

## Data Availability

The datasets used and/or analyzed during the current study are available from the corresponding author on reasonable request.
