# Supplementary material for: The GUSBP11/miR-29c-3p axis facilitates gastric cancer malignancy through ceRNA mechanism
Source: Hereditas. 2026 Aug 12;163:128. doi: 10.1186/s41065-026-00719-1 (PMC13749797; doi:10.1186/s41065-026-00719-1)

Figure 6 F

THBS2 (180kDa)

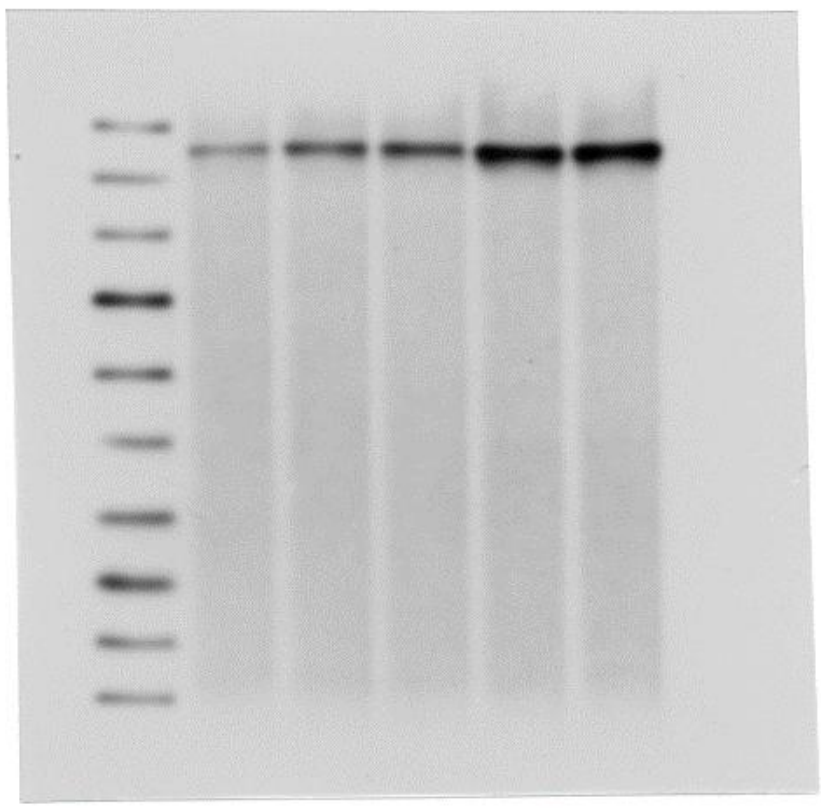

GAPDH (36kDa)

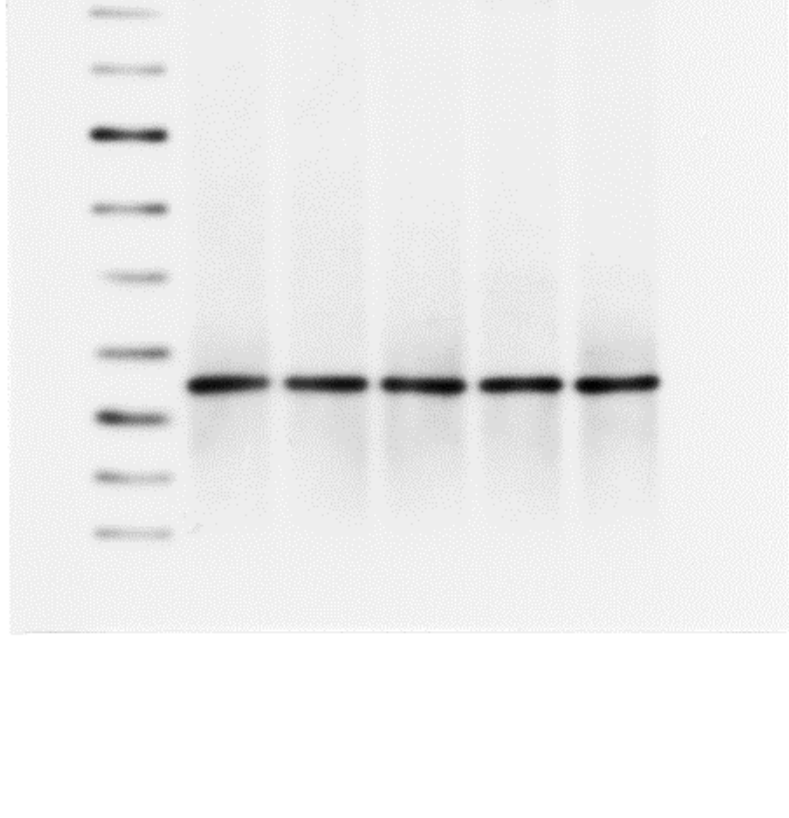

Figure 6G-AGS

THBS2 (180kDa)

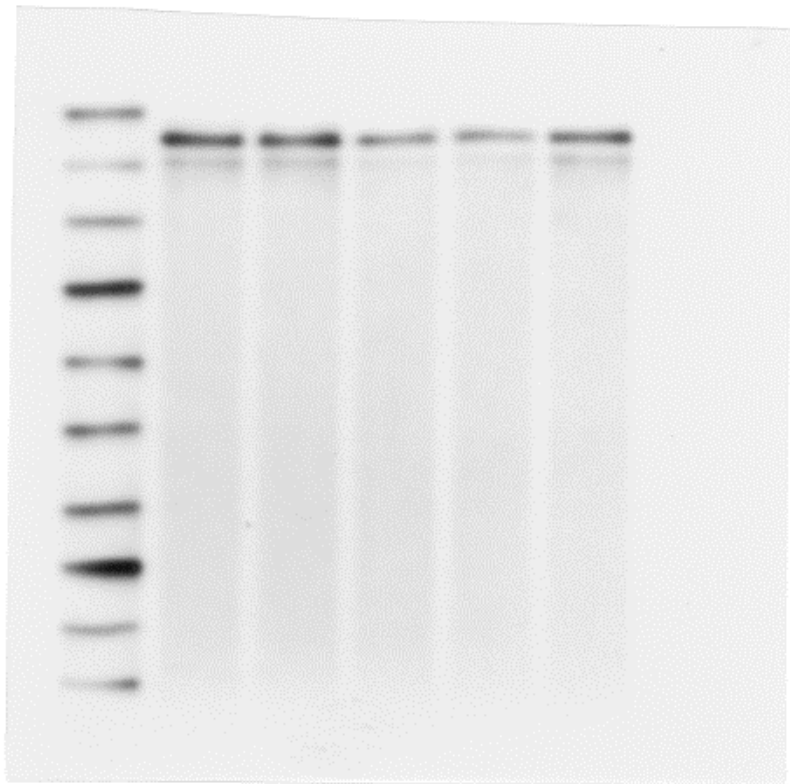

GAPDH (36kDa)

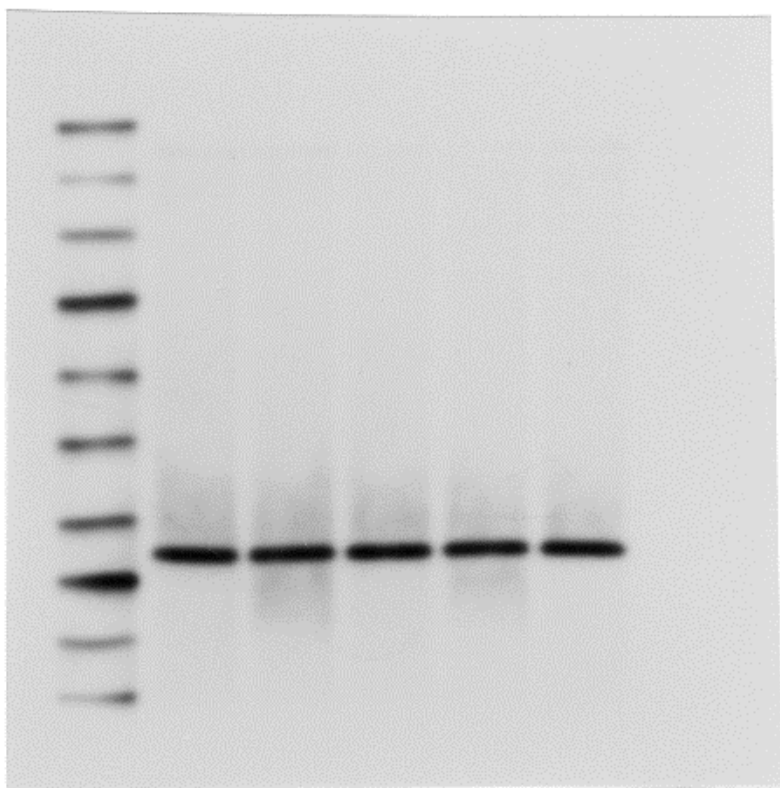

Figure 6G-HGC-27

THBS2 (180kDa)

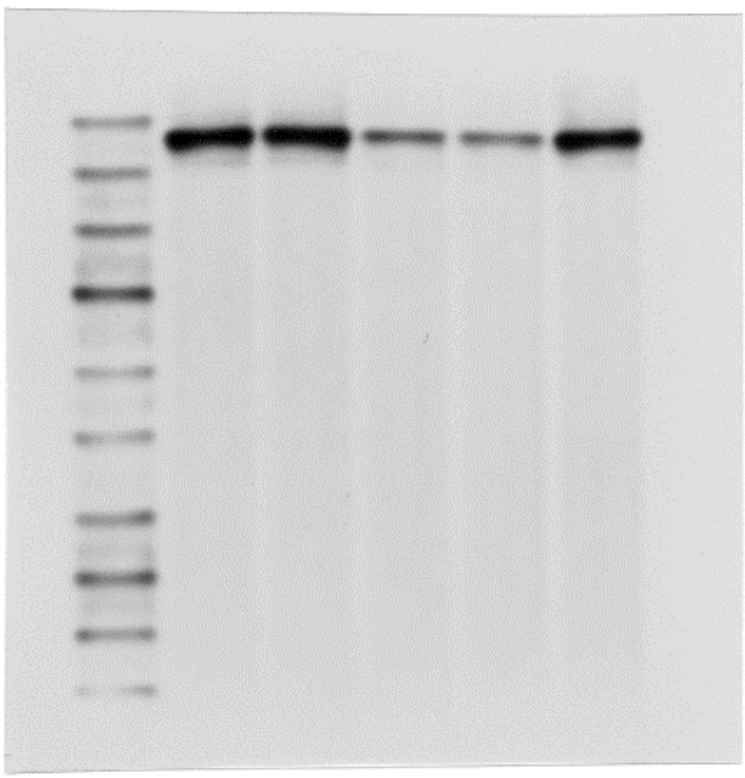

GAPDH (36kDa)

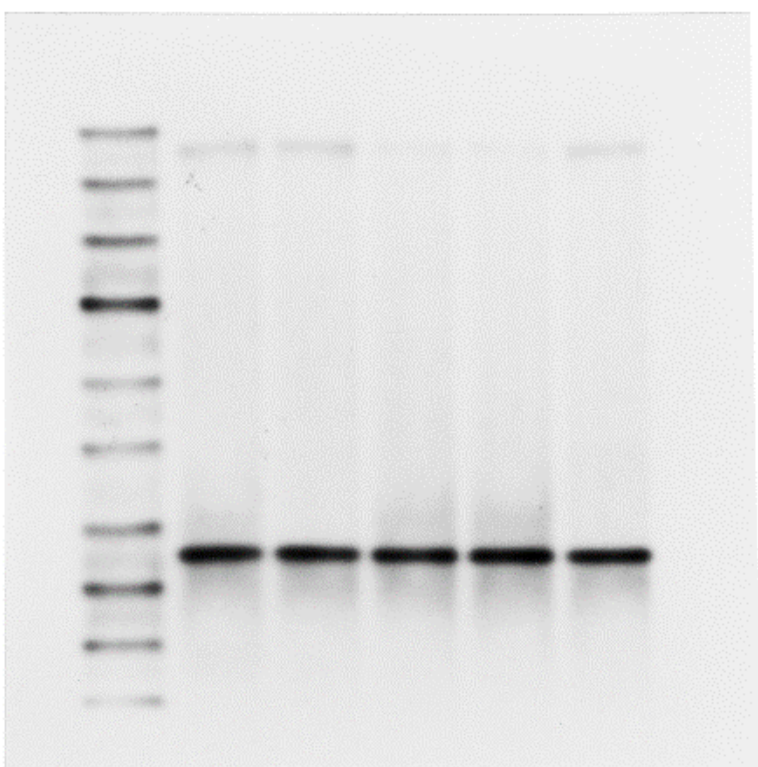

Marker

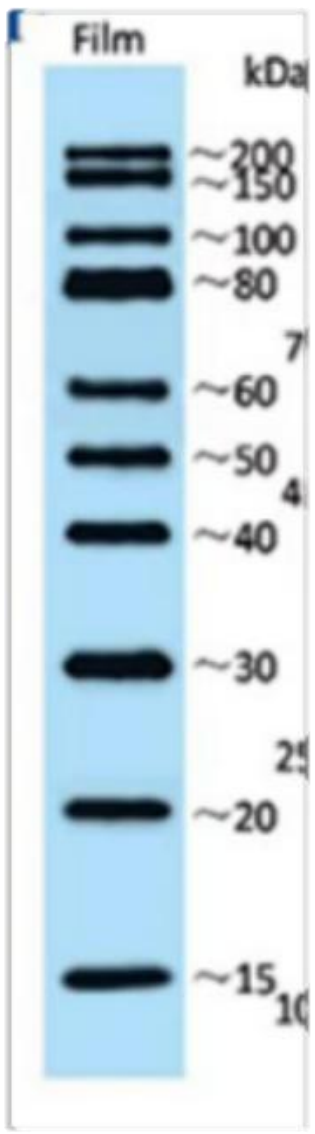

Supplement: Supplementary file 1 [file 41065_2026_719_MOESM1_ESM.pdf]
